# Supplementary material for: Bio-Inspired Aramid Fibers@silica Binary Synergistic Aerogels with High Thermal Insulation and Fire-Retardant Performance
Source: Polymers (Basel). 2022 Dec 28;15(1):141. doi: 10.3390/polym15010141 (PMC9824314; doi:10.3390/polym15010141)
Supplement: Supplementary file 1 [file polymers-15-00141-s001.zip › polymers-2084754-supplementary.pdf]

Supplementary Materials

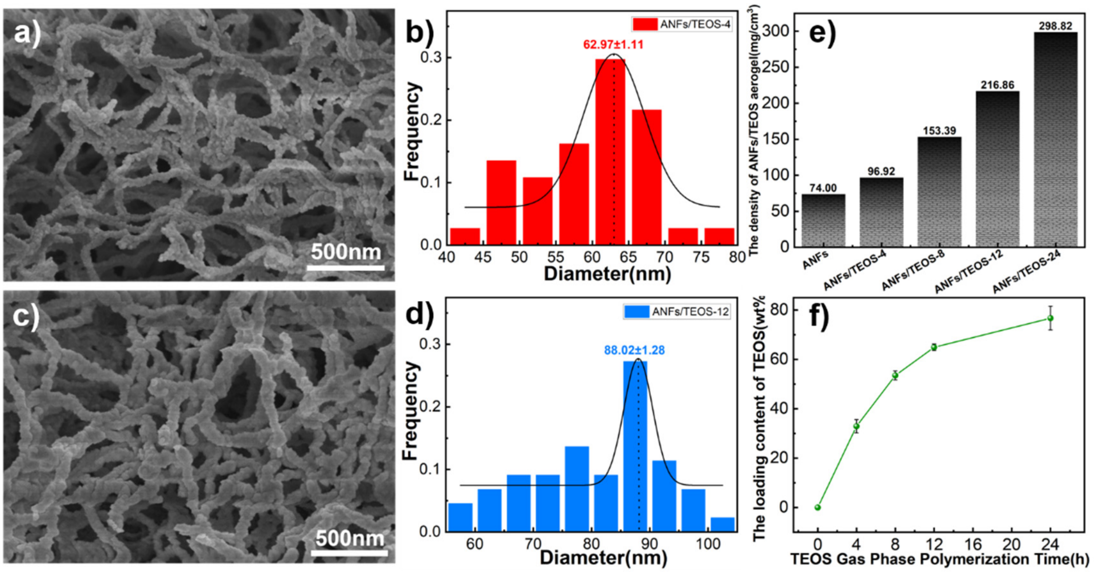

Figure S1. SEM images of aramid fibers@silica aerogel prepared by CVD 4 h a) and 12 h c), together with the histograms of the corresponding nanofibers. e) Mass fraction of silica in ANFs/TEOS aerogel prepared by different CVD times. f) Density of ANFs/TEOS aerogel prepared by different CVD times.

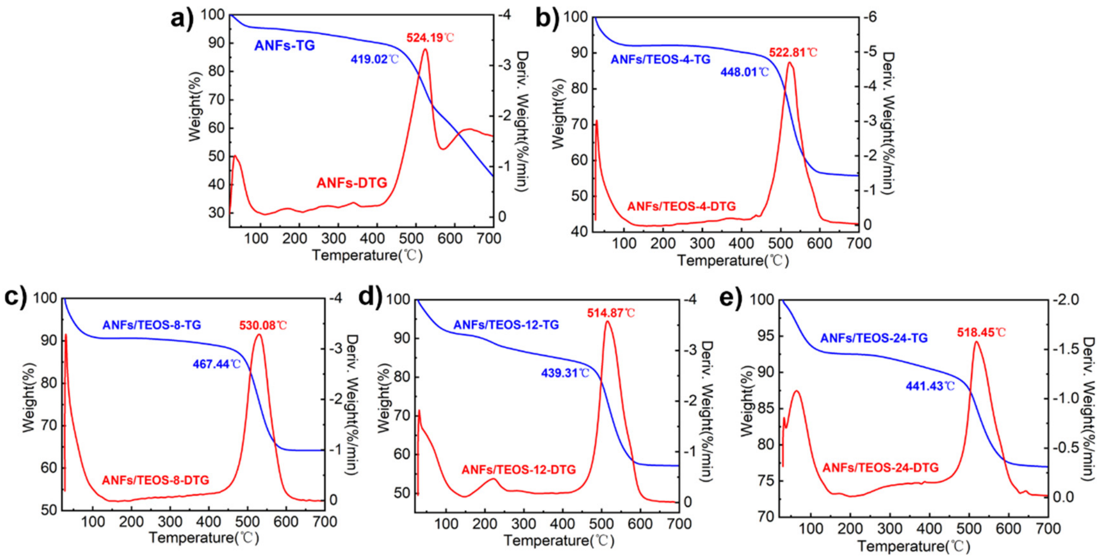

Figure S2. The TG and DTG curves of ANFs a), ANFs/TEOS-4 b), ANFs/TEOS-8 c), ANFs/TEOS-12 d), and ANFs/TEOS-24 e) from 25 ºC to 700 ºC at oxygen atmosphere.

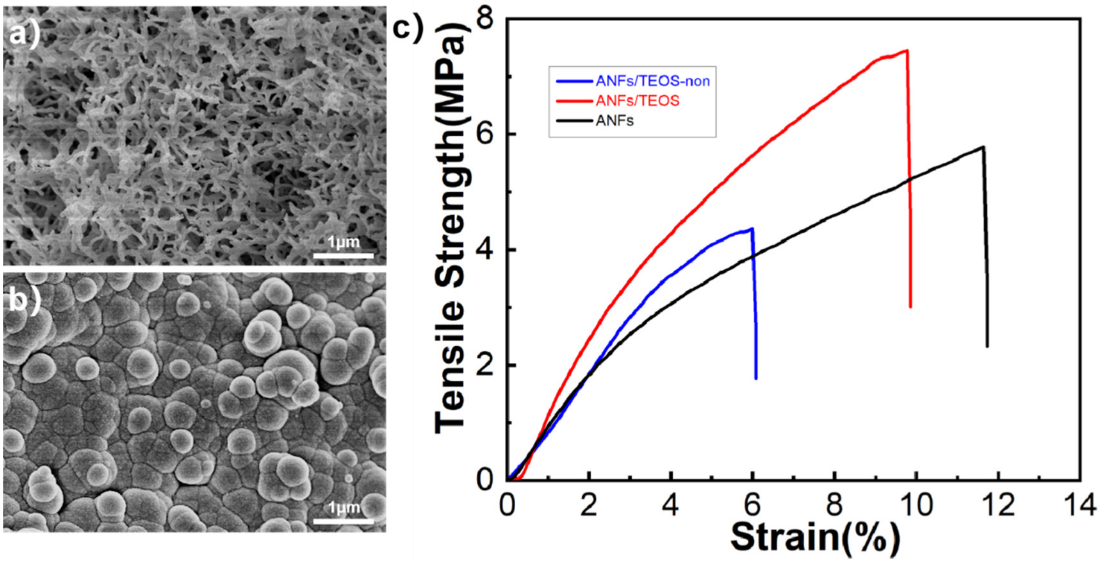

Figure S3. SEM images of ANFs/TEOS aerogel with shell-core structure a) and ANFs/TEOS aerogel with non-putamen structure b). c) Stress-strain curves of ANFs/TEOS aerogel with non-putamen structure (ANFs/TEOS-non), ANFs/TEOS aerogel with shell-core structure (ANFs/TEOS) and ANFs.

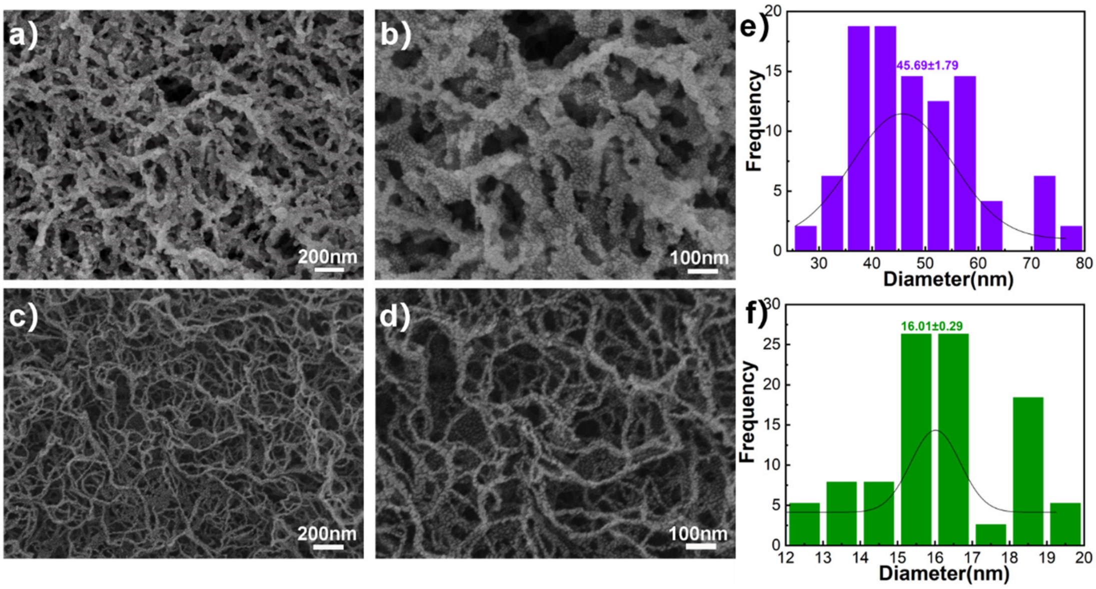

Figure S4. SEM images of ANFs/TEOS-8 after high temperature ablation (a, b), and ANFs after high temperature ablation (c, d), together with the histograms of the corresponding nanofibers.
